# Admission Biomarkers as Predictors of Mortality in Comatose Patients in the Intensive Care Unit: A Retrospective Pilot Study

**DOI:** 10.3390/diagnostics16091388

**Published:** 2026-05-03

**Authors:** Pompiliu Mircea Bogdan, Roxana Elena Bogdan-Goroftei, Alina Plesea-Condratovici, Adina Oana Armencia, Letitia Doina Duceac, Camer Salim, Cristian Gutu, Manuela Arbune, Lavinia-Alexandra Moroianu, Constantin Marinel Vlase, Monica Mihaela Scutariu, Alina Mihaela Calin

**Affiliations:** 1 Doctoral School of Biomedical Sciences, Faculty of Medicine and Pharmacy, Research Center in the Medical-Pharmaceutical Field, “Dunarea de Jos” University of Galati, 47 Domneasca Str., 800008 Galati, Romania; pompiliu.bogdan@ugal.ro (P.M.B.); elenamed84@yahoo.com (R.E.B.-G.); letimedr@yahoo.com (L.D.D.); cristian.gutu@ugal.ro (C.G.); manuela.arbune@ugal.ro (M.A.); lavinia.moroianu@ugal.ro (L.-A.M.); alina.calin@ugal.ro (A.M.C.); 2 “Grigore T. Popa” University of Medicine and Pharmacy Iasi, 16 Universitatii Str., 700115 Iasi, Romania; mihaela.scutariu@umfiasi.ro; 3 Faculty of Medicine, “Ovidius” University of Constanta, 1 Aleea Universității, 900470 Constanța, Romania; salimcamer@yahoo.com; 4 “Dr. Aristide Serfioti” Emergency Military Hospital Galati, 199 Traian Str., 800150 Galati, Romania; constantin.vlase@ugal.ro

**Keywords:** intensive care, mortality, biomarkers, lactate dehydrogenase, APTT, prognosis, critical patients, ROC analysis

## Abstract

**Background**: Intensive care units (ICUs) provide management of critically ill patients requiring continuous monitoring and complex therapeutic interventions. The aim of this study was to analyze the clinical and biological characteristics associated with mortality in patients admitted to the intensive care unit. **Methods**: This retrospective observational study included 108 adult patients admitted to the Anesthesia and Intensive Care Unit of the “Sf. Apostol Andrei” Emergency County Clinical Hospital in Galați, who were in a coma at the time of admission. Demographic data, comorbidities, clinical parameters and biological biomarkers determined at admission were analyzed. Statistical analysis was performed using the SPSS program and included non-parametric tests (Mann–Whitney U), Spearman correlation analysis, multivariate logistic regression and ROC curve analysis to evaluate the predictive performance of biomarkers. **Results**: Hypertension (60.2%) and diabetes mellitus (35.2%) were the most common comorbidities. Comparative analysis revealed significant differences between deceased and surviving patients for several biological parameters, including leukocytes, C-reactive protein, LDH, D-dimers, INR and APTT. In multivariate analysis, LDH (OR = 0.998; *p* < 0.001) and APTT (OR = 0.951; *p* = 0.033) remained independently associated with mortality. ROC analysis revealed good discrimination capacity for LDH (AUC ≈ 0.805) and moderate performance for APTT. **Conclusions**: Determination of LDH and APTT at the time of admission to the ICU may provide useful information for assessing the prognosis of critically ill patients and for early stratification of mortality risk.

## 1. Introduction

Advances in critical care medicine have led to significant improvements in the management of patients with severe and potentially life-threatening conditions. In this context, intensive care units (ICUs) are essential components of hospital systems, designed to care for critically ill patients who require continuous monitoring, ventilatory support, intravenous medication administration and complex therapeutic interventions. Although technological development and progress in knowledge in critical care medicine have contributed to improving the care of critically ill patients, mortality in ICUs remains high and constitutes an important indicator of the performance of health systems. In this context, the identification of factors associated with patient outcome is essential to guiding therapeutic decisions and to estimating prognosis [1,2]. Numerous studies have analyzed various predictors of mortality, such as age, demographic characteristics, delays in admission, creatinine level or Glasgow Coma Scale score [3,4].

Globally, mortality rates in intensive care units vary considerably between regions, reflecting differences in healthcare infrastructure, availability of specialized staff, and the clinical profile of admitted patients. Approximately four million ICU admissions are reported annually in the United States, with mortality rates generally ranging between 8% and 19%. In developed regions such as North America, Oceania, Asia, and Europe, mortality tends to be lower, while higher values have been reported in some areas of South America and the Middle East [5,6].

Higher mortality rates have also been described in several African countries. Studies conducted in Nigeria, Tanzania, and Kenya reported mortality rates ranging from approximately 30% to over 50% [6,7], while research from Ethiopia and Rwanda showed values between approximately 27% and 46%, depending on the characteristics of the studied population and local healthcare resources [4,8,9]. These findings suggest that both clinical factors and healthcare system characteristics may influence outcomes in critically ill patients.

Patients admitted to the ICU in a comatose state represent a particularly severe clinical subgroup, often associated with complex underlying conditions and an increased risk of unfavorable evolution. Altered level of consciousness may occur in various life-threatening conditions, including stroke, traumatic brain injury, severe metabolic imbalance, sepsis, or multiorgan failure. Because of the diversity of causes and the dynamic nature of these conditions, early assessment of prognosis remains difficult, especially at the time of ICU admission.

Severity scores such as APACHE II or SOFA are widely used to estimate mortality risk in critically ill patients. However, these scores require the collection of multiple clinical and laboratory variables, which may not always be immediately available during the initial evaluation [10,11]. For this reason, there is increasing interest in identifying biological parameters that can be obtained rapidly and are already part of routine laboratory testing.

Markers reflecting systemic inflammation, tissue injury, or coagulation abnormalities may provide additional information from the early stages of patient evaluation. Such parameters could support a more rapid estimation of prognosis and may assist clinical decision-making, particularly in situations where therapeutic interventions must be initiated without delay.

In contrast, some of the highest mortality rates in ICUs have been reported in African countries, where studies in Nigeria, Tanzania, and Kenya have reported rates ranging from approximately 30% to over 50% [6,7]. Studies in Ethiopia and Rwanda have reported comparable results, with mortality rates ranging from approximately 27% to 46%, depending on the medical center and population characteristics studied [4,8,9]. Thus, a study conducted in Jordan reported a mortality rate in the ICU of 34%, higher than that observed in most intensive care units in developed countries and close to the levels reported in some developing countries [1]. Another study conducted in Africa on a smaller number of patients and trauma cases revealed a mortality of 29% [4]. Similarly, research conducted in Turkey reported a higher mortality rate of 55% [12], while the multicenter study conducted in Europe indicated a considerably lower mortality rate of 19% [13].

Numerous studies have investigated the factors associated with mortality in patients admitted to the ICU, highlighting the role of clinical and therapeutic variables such as advanced age, diagnosis at admission, need for mechanical ventilation, use of vasopressors, length of stay in the ICU, altered state of consciousness or the presence of acute respiratory distress syndrome [14]. Factors such as anemia, delayed admission to the ICU, or overcrowding in the emergency department (emergency department boarding) have also been associated with less favorable clinical outcomes in critically ill patients, although the results of studies are not always consistent [15].

Identifying factors associated with the evolution of critically ill patients is an essential element for optimizing therapeutic strategies and estimating prognosis. In this context, numerous scoring systems have been developed to assess the severity of the disease and estimate the risk of mortality in intensive care. Among the most widely used prognostic tools are the Acute Physiology and Chronic Health Evaluation (APACHE) and the Sequential Organ Failure Assessment (SOFA), which integrate clinical and physiological variables to estimate the severity of the patient’s condition and the probability of survival. Subsequently, other prognostic models were developed, such as the Mortality Prediction Model (MPM) in the United States and the Simplified Acute Physiology Score (SAPS) in Europe, which were subsequently updated to reflect evolving medical practices and changes in the profile of ICU patients [14,15]. Although these scores are widely used in clinical practice and research, their application requires the collection of a relatively large number of parameters and may be limited in certain clinical settings [8].

In recent years, the interest in identifying simple biological biomarkers that are easy to determine and available in routine clinical practice has increased considerably in critical care medicine. Biochemical markers can reflect important pathophysiological processes, such as systemic inflammation, tissue damage, cellular hypoxia or activation of the coagulation system, all of which are frequently involved in the evolution of critical conditions. The evaluation of these parameters at the time of admission to the intensive care unit could contribute to the early identification of patients at high risk of adverse outcomes. Lactate dehydrogenase (LDH) is an intracellular enzyme involved in cellular energy metabolism and is released into the circulation under conditions of tissue damage, hypoxia or cell destruction. Elevated LDH levels have been associated in numerous studies with disease severity and poor prognosis in various critical pathologies, including sepsis, acute respiratory failure or severe trauma [16,17]. Elevated LDH may reflect the intensity of tissue damage and the degree of systemic metabolic stress, and is considered a nonspecific but useful marker in the evaluation of critically ill patients.

Similarly, coagulation system disorders are a frequent feature of critical conditions and may reflect the complex interaction between systemic inflammation, endothelial dysfunction, and activation of thrombotic mechanisms. Parameters such as activated partial thromboplastin time (APTT) are commonly used to assess the coagulation system and may provide relevant information on hemostatic changes associated with severe pathologies. Coagulation disorders have been described as being associated with adverse outcomes in numerous clinical situations, including sepsis or multiorgan failure [18,19,20].

Although numerous biomarkers have been investigated in the context of critical care medicine, the results reported in the literature remain heterogeneous, and the prognostic utility of some biological parameters determined at the time of admission to the ICU is not yet fully clarified. In this context, the identification of accessible biological markers that are easy to integrate into the initial clinical assessment of critically ill patients remains an important research direction.

The decision to admit and manage patients in intensive care units involves the rapid assessment of the severity of the disease, the prognosis and the available resources. The decision-making process of clinicians in the context of triage and management of critically ill patients is complex and is influenced by numerous clinical and prognostic factors, including neurological status, comorbidities and biological parameters determined at the time of admission [21].

The aim of this study was to describe the clinical and biological characteristics of comatose patients admitted to the Anesthesia and Intensive Care Unit and to assess the relationship between admission laboratory parameters and patient outcomes, particularly mortality. The study started from the hypothesis that certain biological parameters measured upon admission are associated with mortality in comatose patients admitted to the intensive care unit.

## 2. Materials and Methods

### 2.1. Study Design

The study was designed as a retrospective pilot observational study, with the objective of analyzing the clinical and paraclinical characteristics of patients in a coma and hospitalized in the Anesthesia and Intensive Care Unit of the “Sf. Apostol Andrei” Emergency County Clinical Hospital, Galați Romania. The study design allowed the evaluation of demographic, clinical and biological data recorded at the time of hospitalization, as well as the analysis of the relationships between these parameters and the evolution of the patients during hospitalization in the intensive care unit. The data were obtained by analyzing the medical documentation and clinical records of the patients included in the study.

The study was reported in accordance with the STROBE (Strengthening the Reporting of Observational Studies in Epidemiology) guidelines (see Supplementary Table S1).

### 2.2. Participants and Selection Criteria

The study was conducted in December 2025 on a group of 108 adult patients admitted to the Anesthesia and Intensive Care Unit of the “Sf. Apostol Andrei,” Emergency County Clinical Hospital in Galați, who was in a coma at the time of ICU admission.

The diagnosis of coma was based on the information available in the patients’ medical records at the time of admission, including clinical examination findings and physician notes. In practice, patients were considered comatose when they presented with a severely altered level of consciousness, with no eye opening, no purposeful motor response to stimuli, and an inability to follow verbal commands.

In the reviewed medical charts, this diagnosis was supported by the neurological evaluation performed at admission, which typically included descriptions of the level of consciousness, pupillary reactions, motor response, and, in many cases, the need for airway protection or mechanical ventilation. Glasgow Coma Scale (GCS) scores were recorded in some patients, but not consistently across all cases, and therefore were not used as a uniform inclusion criterion.

In addition, the diagnosis was confirmed when terms such as “coma” or “comatose state” were explicitly mentioned in the admission notes, ICU observation charts, or discharge summaries.

The underlying conditions were heterogeneous and reflected common causes of impaired consciousness in critically ill patients, including cerebrovascular events, metabolic disorders, sepsis, and multiorgan failure. Due to the retrospective design and variability in documentation, it was not possible to classify the etiology of coma in a standardized manner for all patients.

Patients were selected retrospectively from data available in medical records and clinical observation sheets during the analyzed period.

The study was conducted in accordance with the principles of the Declaration of Helsinki and approved by the Ethics Committee of the institution (No. 26370/24 November 2025).

Patients over 40 years of age who were comatose at the time of admission to the ICU and for whom relevant clinical and biological data were available in the medical documentation, as well as information on the clinical evolution during hospitalization, were included in the study. Patients with incomplete medical documentation or for whom the data necessary to evaluate the parameters included in the analysis could not be identified were excluded.

Demographic, clinical and biological data recorded at the time of admission to the intensive care unit were collected for each patient. The demographic parameters analyzed included the age and sex of the patients. The main associated comorbidities were also recorded, such as hypertension, diabetes mellitus, atrial fibrillation, chronic heart failure, chronic ischemic heart disease, chronic kidney disease, obesity and history of stroke. Information on the cause of coma was not available in a consistent manner for all patients, as this was a retrospective study based on data recorded in the medical charts. For this reason, it was not possible to classify cases uniformly according to etiology (e.g., structural, metabolic, toxic or infectious).

Clinical assessment upon admission included determination of peripheral oxygen saturation (SpO_2_) and the need for respiratory support. The main biological constants determined during the first laboratory investigations performed upon admission to the ICU were analyzed, including lactate dehydrogenase (LDH), procalcitonin, D-dimers and arterial blood pH, leukocyte count, C-reactive protein, INR and APTT.

The serum lactate dehydrogenase (LDH) value determined at the time of admission to the Intensive Care Unit (ICU) was recorded for each patient based on the first laboratory analyses performed upon admission. LDH was expressed in units per liter (U/L) and was analyzed as a continuous variable.

The evolution of the patients was monitored during hospitalization in the intensive care unit, the main outcome variable analyzed being mortality in the ICU, defined as death occurring during hospitalization in the intensive care unit, compared to the transfer of the patient to other clinical wards in case of favorable evolution.

**Figure 1:**
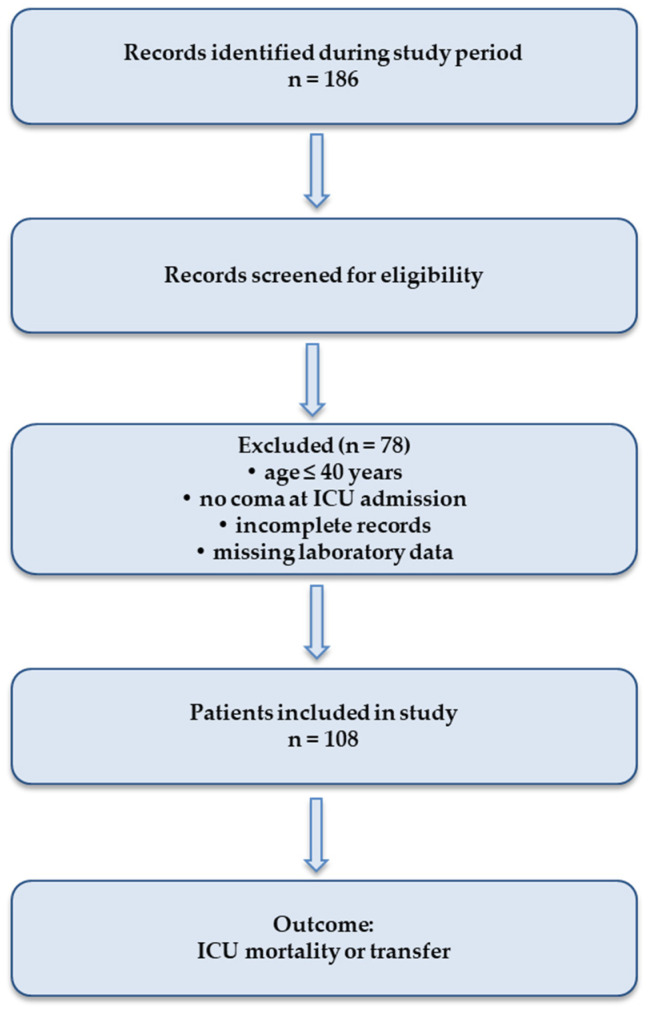
Flow diagram illustrating the process of patient selection and inclusion in the statistical analysis.

The selection of patients included in the study is presented in Figure 1.

### 2.3. Statistical Analysis

Statistical analysis was performed using IBM SPSS Statistics (version 26, IBM Corp., Armonk, NY, USA). The distribution of continuous variables was assessed by the Kolmogorov–Smirnov and Shapiro–Wilk tests. Since most variables showed non-normal distribution, continuous data were expressed as median and interquartile range (IQR), and categorical variables as absolute frequencies and percentages.

Comparison of continuous variables between patient groups (survivors versus deceased) was performed using the nonparametric Mann–Whitney U test, and categorical variables were compared using the χ^2^ (chi-square) test.

Multivariate binary logistic regression analysis was used to identify independent predictors of mortality in intensive care, including variables with clinical relevance and those that presented *p* values < 0.10 in the univariate analysis. The results were expressed as odds ratios (OR) and 95% confidence intervals (95% CI).

The discriminatory capacity of biomarkers for mortality prediction was assessed by receiver operating characteristic (ROC) curve analysis, calculating the area under the curve (AUC). The optimal cut-off value was determined using the Youden index.

A statistical significance level of *p* < 0.05 was considered statistically significant.

## 3. Results

The study group consisted of 108 patients admitted to the Anesthesia and Intensive Care Unit, predominantly male (57.4%). The age distribution did not follow a normal distribution (Shapiro–Wilk test, *p* < 0.001). The median age of the patients was 72 years (IQR = 17), with values ranging from 20 to 99 years. The mean value (68.87 years) was lower than the median, and the negative skewness (−0.93) indicated a leftward asymmetry of the distribution (Table 1).

Comorbidity analysis revealed an increased prevalence of cardiovascular pathologies in the study group. The most common condition identified was hypertension, present in 65 patients (60.2%), followed by diabetes mellitus (38 cases; 35.2%) and chronic heart failure (34 cases; 31.5%). Obesity was observed in 32 patients (29.6%), and chronic ischemic heart disease in 28 patients (25.9%). Chronic kidney disease and atrial fibrillation were each identified in 22 patients (20.4%), while a history of stroke was reported in 10 patients (9.3%) (Table 2).

To assess the impact of clinical characteristics and comorbidities on patient outcome, the association between these variables and mortality recorded in the intensive care unit was analyzed.

Comparative analysis of comorbidities according to patient outcome revealed differences in their distribution between deceased patients and those transferred to the ward. Hypertension was the most common comorbidity in both groups, being identified in 67.6% of deceased patients and in 56.3% of surviving patients. A history of stroke was more common among deceased patients (16.2%) compared to those transferred to the ward (5.6%). The other comorbidities analyzed, including chronic kidney disease, obesity, chronic heart failure, atrial fibrillation, and diabetes mellitus, showed relatively similar distributions between the two groups of patients (Table 3).

Comparative analysis of biological parameters determined at the time of admission to the Anesthesia and Intensive Care Unit revealed statistically significant differences between deceased patients and those transferred to the unit for several inflammatory and coagulation markers. Thus, leukocyte values (*p* = 0.020), C-reactive protein (*p* = 0.005), lactate dehydrogenase—LDH (*p* < 0.001), D-dimer values (*p* = 0.005), INR (*p* = 0.024) and APTT (*p* = 0.009) showed significant differences between the two groups of patients. On the other hand, procalcitonin values (*p* = 0.424), arterial carbon dioxide (*p* = 0.341) and bicarbonate (*p* = 0.482) did not reveal statistically significant differences. Also, the values of arterial pH (*p* = 0.056) and peripheral oxygen saturation—SpO_2_ (*p* = 0.080) showed a trend towards statistical significance (Table 4).

Spearman correlation analysis was performed to evaluate the relationships between the main biological parameters determined at the time of admission to the Anesthesia and Intensive Care Unit. The results revealed the existence of statistically significant correlations between several of the analyzed markers, suggesting complex interactions between inflammatory processes, tissue damage and changes in the coagulation system characteristic of critical conditions (Table 5).

In particular, C-reactive protein values showed a moderate positive correlation with both LDH values (ρ = 0.312; *p* = 0.001) and D-dimer levels (ρ = 0.301; *p* = 0.002). These results indicate that the intensity of the systemic inflammatory process may be associated with the degree of tissue damage and the activation of coagulation mechanisms in critical patients.

LDH also demonstrated a moderate positive correlation with D-dimers (ρ = 0.406; *p* < 0.001), suggesting that processes of cellular damage and tissue hypoxia may be accompanied by activation of the coagulation cascade and intensification of thrombotic processes. This relationship is relevant in the context of severe critical conditions, in which systemic inflammation and endothelial dysfunction may contribute to the development of coagulopathy.

Another important result was the moderate positive correlation observed between INR and APTT (ρ = 0.503; *p* < 0.001), which reflects the interdependence of the parameters used in the evaluation of the coagulation system. Similarly, D-dimers showed significant correlations with INR (ρ = 0.294; *p* = 0.002) and APTT (ρ = 0.259; *p* = 0.007), suggesting the concomitant involvement of thrombotic processes and hemostatic changes in the evolution of critically ill patients.

Regarding acid–base parameters, arterial pH showed significant negative correlations with D-dimers (ρ = −0.238; *p* = 0.013) and INR (ρ = −0.202; *p* = 0.036). These relationships indicate that lower pH values, reflecting a tendency towards metabolic or respiratory acidosis, may be associated with activation of coagulation mechanisms and increased markers of thrombosis. Overall, the correlation analysis highlights that inflammatory markers, tissue damage markers, and coagulation system parameters are interrelated in patients admitted to intensive care. These results support the hypothesis that systemic inflammation, endothelial dysfunction, and activation of hemostatic mechanisms represent interdependent pathophysiological processes involved in the evolution of critical conditions.

To identify independent factors associated with mortality in patients admitted to the Anesthesia and Intensive Care Unit, a multivariate logistic regression analysis was performed, including in the model the values of biomarkers determined at the time of admission to the ICU that showed significant differences or trends towards statistical significance in the previous comparative analysis. The variables introduced into the model were LDH, C-reactive protein, D-dimers, INR, APTT, arterial blood pH and peripheral oxygen saturation (SpO_2_).

The Omnibus test of the model coefficients revealed that the logistic regression model is statistically significant (χ^2^ = 41.350; df = 7; *p* < 0.001), indicating that the variables included in the model contribute significantly to the prediction of patient mortality (Table 6).

The evaluation of the explanatory power of the model revealed a Nagelkerke R^2^ value of 0.453, suggesting that approximately 45.3% of the mortality variability can be explained by the variables included in the model. Also, the value of −2 Log Likelihood = 92.817 indicates a good fit of the model to the analyzed data (Table 7).

The Hosmer–Lemeshow test did not reveal significant differences between the observed values and those estimated by the model (χ^2^ = 8.683; df = 8; *p* = 0.370), suggesting good calibration and adequacy of the logistic regression model (Table 8).

Within the multivariate model, two variables remained significantly associated with mortality in the ICU. Thus, LDH values at ICU admission demonstrated a significant independent association with mortality (OR = 0.998; 95% CI: 0.997–0.999; *p* < 0.001), suggesting that this biochemical marker reflects the degree of tissue damage and the severity of systemic pathological processes. Also, APTT values remained significantly associated with patient outcome (OR = 0.951; 95% CI: 0.908–0.996; *p* = 0.033), highlighting the role of changes in the coagulation system in the prognosis of critically ill patients. In contrast, the other variables included in the model, namely C-reactive protein, D-dimers, INR, arterial pH and SpO_2_, did not demonstrate a significant independent association with mortality after adjustment for the other factors included in the analysis (Table 9).

Overall, the logistic regression results suggest that tissue damage markers and coagulation parameters assessed at the time of admission may contribute to estimating the risk of mortality among critically ill patients, with LDH and APTT emerging as independent factors associated with adverse outcome. These results highlight the importance of integrated assessment of biological markers at admission to the intensive care unit, in order to early identify high-risk patients and optimize therapeutic management strategies.

The results of the multivariate logistic regression model revealed that LDH and APTT were the only biological parameters that remained independently associated with mortality in patients admitted to the intensive care unit.

To evaluate the diagnostic performance of lactate dehydrogenase (LDH) values determined at the time of admission to the Anesthesia and Intensive Care Unit in predicting mortality, ROC (Receiver Operating Characteristic) curve analysis was performed. This revealed an Area Under the Curve (AUC) of approximately 0.805, a value that indicates a good discrimination capacity of LDH for predicting mortality among critically ill patients. According to the ROC curve interpretation criteria, an AUC value between 0.8 and 0.9 reflects good diagnostic accuracy, suggesting that the analyzed marker has an adequate capacity to differentiate patients with a high risk of unfavorable evolution from those with a better prognosis.

**Figure 2:**
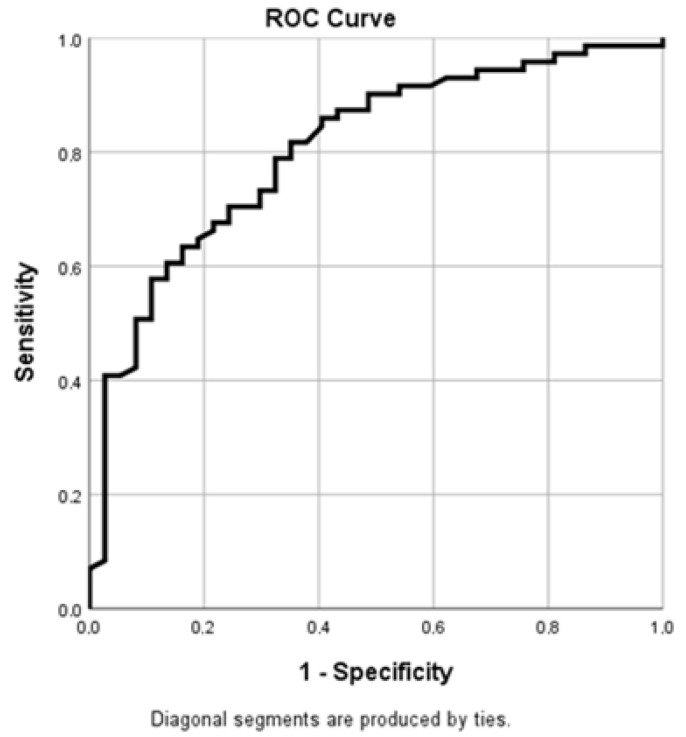
ROC curve of LDH at ICU admission for predicting ICU mortality.

From a clinical perspective, these results indicate that elevated LDH levels determined upon admission to the intensive care unit may reflect a higher degree of tissue damage and systemic metabolic stress, phenomena frequently encountered in severe critical conditions. Thus, LDH may be a useful marker in the initial assessment of patient severity and in the stratification of mortality risk. Integrating this parameter into the clinical and biological evaluation of patients admitted to the ICU could contribute to the early identification of high-risk patients and to the optimization of monitoring and therapeutic management strategies (Figure 2).

In this analysis, the Area Under the Curve (AUC) is an indicator of the discriminative performance of the analyzed marker.

These results suggest that LDH values determined at admission to the intensive care unit may be useful in the prognostic assessment of critically ill patients. Elevated levels of this marker may reflect the degree of tissue damage and the severity of systemic pathological processes, thus contributing to the identification of patients at high risk of mortality and to the optimization of monitoring and therapeutic management strategies.

These results support the potential utility of LDH determination at the time of admission to the intensive care unit as a simple and easily accessible biological marker for early risk assessment in critically ill patients (Table 10).

The receiver operating characteristic (ROC) curve analysis for APTT values revealed an area under the curve (AUC) of 0.346, reflecting a reverse bias of the test direction. After direction correction, the corresponding AUC value is approximately 0.654, suggesting a moderate discriminatory capacity of APTT in predicting mortality in critically ill patients.

**Figure 3:**
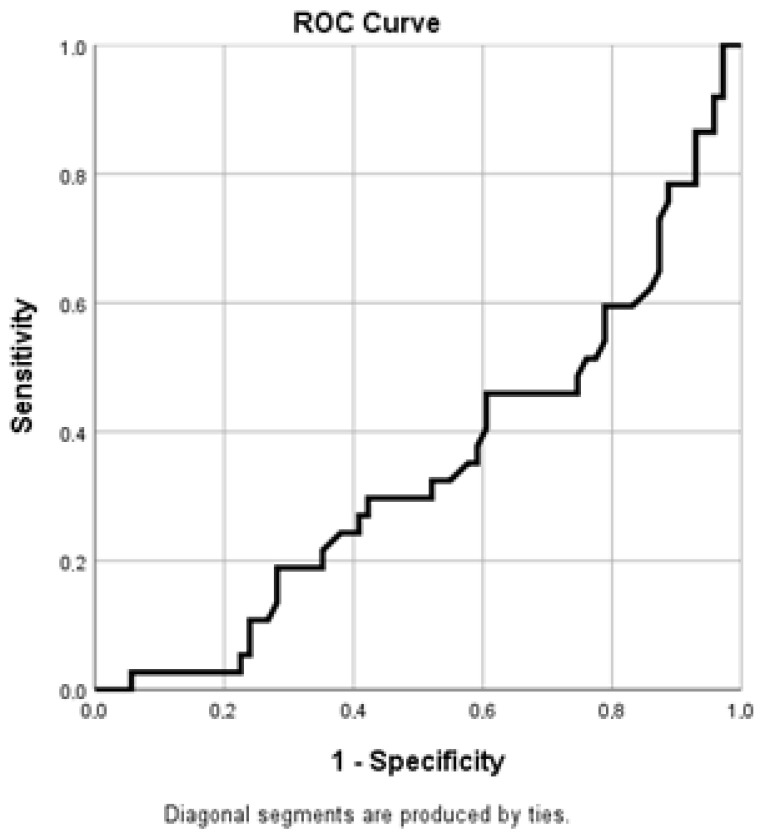
ROC curve of APTT at ICU admission for predicting ICU mortality.

These results indicate that changes in coagulation parameters, reflected by APTT values determined at the time of admission, can provide useful information on the risk of adverse outcome, although the discriminatory performance of this marker remains lower compared to other biomarkers analyzed (Figure 3).

To assess the ability of biological markers to discriminate between deceased and surviving patients, receiver operating characteristic (ROC) curve analysis was performed for LDH and APTT. The optimal cut-off values were determined using the Youden index, and the discriminative performance of the analyzed markers is presented in Table 11. The analysis indicated an optimal cut-off value for LDH of approximately 742.5 U/L, corresponding to a sensitivity of 48.6% and a specificity of 9.9%. In the case of APTT, the optimal cut-off value was estimated at approximately 27.7 s, with a sensitivity of 56.8% and a specificity of 21.1%.

The results of the ROC analysis revealed that LDH determined at the time of admission to the intensive care unit has a good ability to discriminate mortality, with an area under the curve (AUC) of approximately 0.805. For APTT, the corrected AUC value was approximately 0.654, suggesting moderate discrimination. Although the specificity values were relatively low, these results indicate that the determination of LDH and APTT at the time of admission to the ICU may contribute to early risk stratification in critically ill patients, facilitating the identification of patients with a high probability of adverse outcome from the early stages of clinical evaluation.

**Figure 4:**
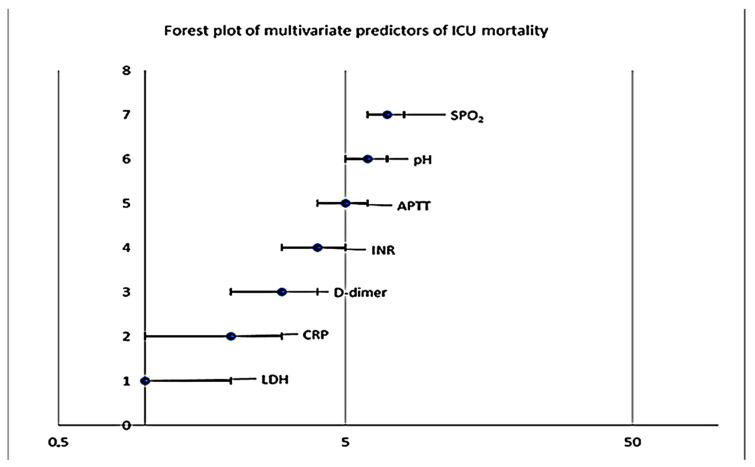
Forest plot of laboratory predictors of ICU mortality.

To evaluate the predictors of mortality in patients admitted to the Anesthesia and Intensive Care Unit, a forest plot of the biological parameters included in the multivariate logistic regression model was performed. This graphic representation illustrates the odds ratios (OR) and 95% confidence intervals (CI95%) for each variable analyzed. The vertical line representing OR = 1 indicates the absence of an association between the analyzed variable and mortality. The analysis of Figure 4 shows that most of the variables included in the model have confidence intervals that intersect the OR = 1 line, which indicates that they are not independently associated with mortality after adjusting for the other factors included in the model. Thus, the values of C-reactive protein (CRP), D-dimers, INR, arterial pH and peripheral oxygen saturation (SpO_2_) did not demonstrate a statistically significant association with mortality in the multivariate model.

In contrast, two variables stand out because their confidence intervals do not intersect the reference line, suggesting an independent association with mortality in critically ill patients. Thus, LDH values at ICU admission demonstrated a significant independent association with mortality (OR = 0.998; 95% CI: 0.997–0.999; *p* < 0.001), suggesting that this biochemical marker reflects the degree of tissue damage and the severity of systemic pathological processes. Also, APTT values remained significantly associated with patient outcome (OR = 0.951; 95% CI: 0.908–0.996; *p* = 0.033), highlighting the role of coagulation system alterations in the prognosis of critically ill patients.

The other variables included in the model, namely C-reactive protein, D-dimers, INR, arterial pH and peripheral oxygen saturation (SpO_2_), did not demonstrate a significant independent association with mortality after adjustment for the other factors included in the analysis (Table 12).

Overall, the results of the logistic regression model indicate that LDH and APTT determined at the time of admission to the intensive care unit may constitute independent factors associated with mortality, suggesting their usefulness in the initial prognostic assessment of critically ill patients.

A particular aspect of the figure is represented by the very wide confidence interval for arterial pH, which suggests a high variability of this parameter within the analyzed group and indicates that the estimate of its effect on mortality is statistically unstable. This situation can be explained by the variability of pH values in critical conditions and by the relatively limited size of the analyzed sample.

Overall, the forest plot highlights that LDH and APTT are the only biological parameters that remain independent predictors of mortality in the ICU in the multivariate model analyzed. The graphic representation facilitates the rapid interpretation of the logistic regression results and highlights the potential role of tissue damage markers and coagulation parameters in assessing the prognosis of critically ill patients.

Table 12 summarizes the results of the univariate analysis and multivariate logistic regression, providing an overview of the clinical and biological factors evaluated in relation to the evolution of the patients included in the study.

In the initial stage, the univariate analysis highlighted a series of biological parameters that showed significant differences between deceased and surviving patients. Thus, leukocyte values (*p* = 0.020), C-reactive protein (*p* = 0.005), LDH (*p* < 0.001), D-dimer (*p* = 0.005), INR (*p* = 0.024) and APTT (*p* = 0.009) demonstrated significant associations with mortality in patients admitted to the ICU. Also, arterial pH values (*p* = 0.056) and peripheral oxygen saturation (SpO_2_) (*p* = 0.080) showed trends towards statistical significance, being subsequently included in the multivariate analysis.

Regarding the comorbidities analyzed, history of stroke showed a trend towards association with mortality (*p* = 0.072), but this relationship did not reach the threshold of statistical significance. The other comorbidities assessed, including chronic kidney disease, obesity, chronic ischemic heart disease, chronic heart failure, atrial fibrillation, hypertension, and diabetes mellitus, did not demonstrate a significant association with mortality in the univariate analysis.

To identify independent factors associated with mortality, relevant variables from the univariate analysis were included in a multivariate logistic regression model. The results of this analysis revealed that only two variables remained significantly associated with patient mortality: LDH values and APTT values determined at the time of admission to the ICU.

Thus, LDH demonstrated a significant independent association with mortality (OR = 0.998; 95% CI: 0.997–0.999; *p* < 0.001), suggesting that this biochemical marker reflects the severity of tissue damage and the intensity of systemic pathological processes present in critically ill patients. LDH is known as an indicator of cellular damage and tissue hypoxia, and elevated values may reflect the degree of metabolic and inflammatory damage associated with severe critical conditions.

Likewise, APTT values remained independently associated with mortality (OR = 0.951; 95% CI: 0.908–0.996; *p* = 0.033), highlighting the role of changes in the coagulation system in the evolution of patients admitted to intensive care. Hemostatic disorders are frequently encountered in the context of critical pathologies and may reflect complex processes of systemic inflammation, endothelial dysfunction and activation of the coagulation cascade.

In contrast, the other variables included in the multivariate model, namely C-reactive protein, D-dimers, INR, arterial pH and SpO_2_, did not demonstrate a significant independent association with mortality after adjustment for the other parameters analyzed. This result suggests that their influence on the evolution of patients may be mediated by other clinical or biological factors, or may reflect complex interactions between different pathophysiological mechanisms involved in critical conditions. Overall, the analysis of mortality predictors highlights the importance of biological markers determined at the time of admission to the ICU for assessing the prognosis of critically ill patients. Early identification of biological parameters independently associated with mortality may contribute to improving risk stratification and optimizing monitoring and therapeutic management strategies in intensive care units.

The results obtained show that several biological parameters determined at the time of admission to the Anesthesia and Intensive Care Unit differ between deceased and surviving patients. Multivariate analysis revealed that LDH and APTT remain independently associated with mortality, suggesting that markers of tissue damage and changes in coagulation parameters may reflect the severity of the clinical condition at the time of admission to the ICU.

## 4. Discussion

The results of the present study highlight the potential role of biological parameters determined at the time of admission to the Anesthesia and Intensive Care Unit in assessing the prognosis of critically ill patients. The analysis aimed to identify factors associated with mortality in the ICU, with a focus on inflammatory markers and coagulation system parameters, frequently used in current clinical practice.

A predominance of male patients was observed in the analyzed group. This observation is consistent with data reported in the specialized literature, which show that men are more frequently admitted to intensive care units compared to women, especially in the context of cardiovascular or metabolic pathologies. Gender differences in ICU admissions have been described in several epidemiological studies and can be explained by the higher prevalence of cardiovascular and metabolic risk factors among the male population [22,23,24].

Also, the relatively high median age of the patients included in the study reflects the trend observed globally of increasing the proportion of elderly patients admitted to intensive care, a category characterized by a high prevalence of comorbidities and a reduced physiological reserve. Recent studies dedicated to the epidemiology of critically ill patients have shown that advanced age remains associated with a higher risk of mortality in intensive care, even after adjusting for severity scores, highlighting the increased vulnerability of this category of patients [25,26,27,28].

The analysis of comorbidities revealed an increased frequency of cardiovascular pathologies, especially hypertension, chronic heart failure and chronic ischemic cardiopathy. These results are consistent with data reported in recent epidemiological studies on the population of critically ill patients, where cardiovascular diseases are described as being among the most frequent comorbidities associated with admission to the ICU [29]. The presence of these diseases may influence the evolution of patients by reducing the functional reserve of organs and by increasing susceptibility to systemic complications. Recent epidemiological studies indicate the same thing, namely that cardiovascular diseases are present in a significant proportion of patients admitted to intensive care units and may influence the clinical evolution by reducing the functional reserve and increasing susceptibility to multiorgan dysfunction [30,31]. The presence of these diseases may contribute to the worsening of the hemodynamic and metabolic status of the critically ill patient, thus influencing the evolutionary prognosis.

Comparison of the distribution of comorbidities according to patient outcome showed that a history of stroke was more common in deceased patients, although this association did not reach statistical significance. Similar trends have been reported in other clinical studies, which have shown that patients with a history of cerebrovascular pathology may be at higher risk of adverse outcome in the context of critical conditions, especially due to neurological fragility and associated cardiovascular comorbidities [32,33,34,35,36]. In this context, a history of cerebrovascular disease can be considered an indicator of systemic vulnerability, even if the relationship with mortality is not always statistically significant in relatively small cohorts.

Regarding the biological parameters analyzed at the time of admission, the study results revealed significant differences between deceased and surviving patients for several inflammatory and coagulation markers. The values of leukocytes, C-reactive protein, LDH, D-dimers, INR and APTT were significantly different between the two groups of patients. These results suggest the important role of systemic inflammation and hemostatic disorders in the evolution of critical conditions. In the literature, inflammatory markers and coagulation parameters are frequently described as indicators of disease severity and prognosis in various critical pathologies, including sepsis or acute respiratory failure [37,38,39,40]. Recent literature emphasizes that inflammatory markers and coagulation system parameters are closely interconnected in the evolution of critical patients, being involved in complex mechanisms of immune activation, endothelial dysfunction and thrombo-inflammation [39,41,42]. In recent cohorts of patients with sepsis or other severe pathologies, elevated levels of inflammatory and coagulation markers at admission were associated with higher mortality and more severe clinical course [43].

Multivariate logistic regression analysis performed in this study revealed that only two variables remained independently associated with mortality: LDH values and APTT values determined at the time of admission to the ICU. LDH is a nonspecific biochemical marker of tissue damage and cellular metabolism, being released into the circulation in the context of cellular destruction or tissue hypoxia. Although LDH values were higher in patients who died, the logistic regression coefficient generated an odds ratio very close to 1. However, it should be borne in mind that LDH was entered into the model as a continuous variable, and the odds ratio reflects the change in risk for a single unit increase in the biomarker value. In the case of LDH, whose values can vary over wide ranges, a one-unit increase has a minimal impact on the risk estimate, which explains the OR value close to 1. Interpretation must therefore be made in the context of the wider variations in the biomarker. Numerous studies published in recent years have shown that elevated LDH values are associated with disease severity and mortality in various critical pathologies, including sepsis, acute respiratory failure, or severe stroke [44,45,46]. Henry et al. have shown that elevated LDH levels are associated with unfavorable outcome and increased mortality in critically ill patients [44,47]. Zhang et al. also reported that LDH is a useful marker for assessing the severity and prognosis in various severe systemic diseases [48]. Also, biomarkers derived from LDH, such as the LDH–albumin ratio, have been proposed as useful prognostic indicators in critical care medicine, suggesting that this marker may simultaneously reflect tissue damage and systemic metabolic disorders [48].

Similarly, APTT demonstrated an independent association with mortality in the multivariate model. Coagulation system disorders are frequently encountered in the context of critical conditions and reflect the complex activation of inflammatory and thrombotic mechanisms. Coagulopathy associated with critical conditions has been described in numerous studies as an important factor in the evolution of patients with sepsis or other severe pathologies [42,49]. Changes in coagulation parameters, including APTT prolongation, may reflect the consumption of coagulation factors and the activation of the systemic inflammatory system. An important result of the study is represented by the good discrimination capacity of LDH in the prediction of mortality, highlighted by ROC curve analysis. The value of the area under the curve (AUC ≈ 0.805) indicates a good diagnostic performance of this marker in differentiating patients at high risk of unfavorable evolution from those with a more favorable prognosis. According to the criteria used in the interpretation of ROC curves, an AUC value between 0.8 and 0.9 reflects good diagnostic accuracy [50]. Similar results have been reported in other recent studies that evaluated the role of LDH as a prognostic marker in critical pathologies.

Although LDH and APTT were statistically associated with mortality, their sensitivity and specificity were relatively low. For this reason, these parameters should be interpreted with caution and cannot be considered reliable predictors when used alone. Their usefulness may be greater when evaluated together with clinical data and other laboratory findings.

The graphical representation of the results of the logistic regression by forest plot allowed a synthetic visualization of the effect of each analyzed variable on mortality. This analysis confirms that most of the biological parameters included in the model did not demonstrate a significant independent association after adjustment for other factors. In contrast, LDH and APTT emerged as independent predictors of mortality in the analyzed group.

From a clinical perspective, the results of this study suggest that the evaluation of biological markers at the time of admission to the ICU may contribute to a more rapid assessment of the severity of the patient’s condition. Easily accessible parameters, such as LDH and APTT, could be integrated into the initial assessment of critical patients for the early identification of those at high risk of unfavorable evolution.

The interpretation of these findings should also consider that detailed information on the cause of coma and the extent of brain injury was not consistently available. As a result, these factors could not be included in the analysis and may have influenced the observed associations between biomarkers and mortality.

Overall, the results of the present study emphasize the importance of biological markers determined at admission in assessing the prognosis of critical patients. However, the interpretation of these results must be made in the context of the study’s limitations, including the relatively small sample size and the observational nature of the analysis. Future studies, conducted on larger groups of patients and in different clinical contexts, could contribute to validating these results and integrating biological markers into more complex prognostic models used in intensive care practice.

### 4.1. Limitations of the Study

The study presented several limitations. First, the relatively small sample size may limit the statistical power of the study and the ability to identify more subtle associations between the investigated variables and mortality in patients admitted to the intensive care unit. A larger number of patients could allow a clearer assessment of the relationships between biological parameters and clinical evolution, as well as a greater stability of the statistical estimates.

Second, the study was conducted in a single center, which may influence the generalizability of the results. The characteristics of the patient population, the therapeutic protocols applied, and the resources available within an intensive care unit may vary significantly between different institutions or health systems. For this reason, the results obtained in this study should be confirmed by multicenter research that includes more diverse patient populations.

Another important limitation is the observational nature of the study. Although the statistical analysis allowed the identification of associations between biological parameters determined at admission and patient mortality, the observational design does not allow the establishment of a direct causal relationship between these variables and clinical evolution. Therefore, the results should be interpreted in terms of statistical associations and not cause–effect relationships.

In addition, the analysis was based exclusively on the values of biological parameters determined at the time of admission to the intensive care unit. The dynamic evolution of these markers during hospitalization was not evaluated. In clinical practice, serial monitoring of some biological parameters, such as inflammatory or coagulation markers, may provide additional information on the patient’s evolution and response to treatment. The lack of these dynamic data may limit the ability to fully evaluate the prognostic role of the analyzed biomarkers.

Other problem that should be mentioned is the absence of severity scores commonly used in the assessment of critically ill patients, such as the Glasgow Coma Scale (GCS), Sequential Organ Failure Assessment (SOFA) or Acute Physiology and Chronic Health Evaluation II (APACHE II). These scores are commonly used to assess the severity of the disease and to estimate the prognosis in intensive care units. In the present study, their inclusion in the statistical analysis was not possible for all patients due to the retrospective nature of the research and the lack of complete records in the clinical documentation. This aspect may represent a limitation in the interpretation of the results. This aspect limits the possibility of directly comparing the predictive performance of the investigated biomarkers with established clinical severity scores.

Another aspect that should be mentioned is the lack of complete clinical data, which is inherent to the retrospective design of the study. Glasgow Coma Scale (GCS) scores were not available for all patients, being recorded in approximately 37% of cases. Similarly, information regarding the cause of coma was inconsistent, and in more than half of the cases it could not be clearly identified from the medical records.

These limitations may have influenced the results. Patients with more complete documentation were likely those more closely monitored or with more severe clinical conditions, which may introduce a certain degree of bias. At the same time, the absence of standardized data on coma severity and etiology limits the possibility of accounting for important clinical factors known to influence prognosis.

For this reason, the results should be interpreted with caution, as some of the observed associations may be influenced by clinical variables that were not included in the analysis.

An important limitation is the lack of detailed information on the extent of brain injury and the underlying cause of coma. Because of the retrospective design and the variability of the medical records, these data were not consistently available and could not be included in the analysis. Since these factors are known to strongly influence patient outcomes, this should be taken into account when interpreting the results.

Biological variability represents another limitation of the study, especially in the context of critical conditions. The values of certain markers, such as arterial pH or inflammatory markers, can vary significantly depending on the time of collection, the therapeutic interventions applied or the rapid evolution of the patient’s clinical status. This variability may influence the estimation of the effect of these parameters on mortality.

A further limitation of the study is the absence of detailed information on the cause of coma, as the retrospective design did not allow a clear classification of cases according to structural or metabolic origin. This should be considered when interpreting the relationship between comorbidities, laboratory parameters and clinical outcome.

Finally, the exclusive use of biological markers in the assessment of prognosis does not fully reflect the complexity of critical conditions. The evolution of patients admitted to intensive care is influenced by a multitude of factors, including the clinical characteristics of the patient, comorbidities, the type of acute pathology, therapeutic interventions and the individual response to treatment. Therefore, biological markers should be interpreted in the context of the overall clinical assessment of the patient.

### 4.2. Future Research Directions

The results of this study suggest the need for further research to confirm the role of biomarkers in assessing the prognosis of critically ill patients. Future studies conducted on larger samples and in multiple centers could help validate the identified associations and increase the generalizability of the results. Also, the analysis of the dynamic evolution of biomarkers during hospitalization could provide additional information on disease progression and response to treatment. The integration of biomarkers with clinical parameters and severity scores used in intensive care could help develop more accurate prognostic models for patient risk stratification.

## 5. Conclusions

The results of this study highlight the potential role of biomarkers determined at the time of admission in assessing the prognosis of critically ill patients. The analysis showed that LDH and APTT values were independently associated with mortality in the multivariate model, suggesting the usefulness of these markers in identifying patients at high risk of adverse outcomes. Also, ROC curve analysis revealed a good discriminatory capacity of LDH in predicting mortality. Overall, the assessment of biomarkers at the time of admission to the ICU may contribute to risk stratification and optimize the management of critically ill patients. However, additional studies, conducted on larger samples and in multiple centers, are needed to confirm these results and to integrate biomarkers into more complex prognostic models.

## Figures and Tables

**Table 1:** Distribution of patients by age.

			Statistic	Std. Error
years	Mean		68.87	1.388
	95% Confidence Interval for Mean	Lower Bound	66.12	
		Upper Bound	71.62	
	5% Trimmed Mean		69.62	
	Median		72.00	
	Variance		208.114	
	Std. Deviation		14.426	
	Minimum		20	
	Maximum		99	
	Range		79	
	Interquartile Range		17	
	Skewness		−0.930	0.233
	Kurtosis		1.364	0.461

**Table 2:** Prevalence of comorbidities in the study group.

Comorbidity	n	%
Hypertension	65	60.2
Diabetes mellitus	38	35.2
Chronic heart failure	34	31.5
Obesity	32	29.6
Chronic ischemic heart disease	28	25.9
Chronic kidney disease	22	20.4
Atrial fibrillation	22	20.4
Stroke	10	9.3

**Table 3:** Distribution of comorbidities according to ICU outcome.

Comorbidity	Death n (%)	Survived n (%)	Total
Stroke	6 (16.2)	4 (5.6)	10
Chronic kidney disease	5 (13.5)	17 (23.9)	22
Obesity	8 (21.6)	24 (33.8)	32
Chronic ischemic heart disease	5 (13.5)	23 (32.4)	28
Chronic heart failure	10 (27.0)	24 (33.8)	34
Atrial fibrillation	7 (18.9)	15 (21.1)	22
Hypertension	25 (67.6)	40 (56.3)	65
Diabetes mellitus	10 (27.0)	28 (39.4)	38

**Table 4:** Mann–Whitney U test for laboratory parameters at ICU admission according to patient outcome.

Test Statistics	Leukocyte Count at ICU Admission	C-Reactive Protein at ICU Admission	Procalcitonin at ICU Admission	LDH at ICU Admission	D-Dimer at ICU Admission	INR at ICU Admission	APTT at ICU Admission	Arterial Blood pH at ICU Admission	SpO_2_ at ICU Admission	Arterial CO_2_ at ICU Admission	Bicarbonate at ICU Admission
Mann–Whitney U	926.5	809.0	1059.0	513.0	877.5	966.0	908.0	1019.0	1043.5	1166.5	1205.0
Wilcoxon W	1592.5	1475.0	1654.0	1216.0	1580.5	1669.0	1611.0	3575.0	3599.5	1869.5	3761.0
Z	−2.318	−2.837	−0.800	−5.182	−2.826	−2.250	−2.625	−1.909	−1.750	−0.952	−0.703
Asymp. Sig. (2-tailed)	0.020	0.005	0.424	<0.001	0.005	0.024	0.009	0.056	0.080	0.341	0.482

**Table 5:** Spearman correlation analysis between laboratory parameters at ICU admission.

Spearman’s Rho		C-Reactive Protein at ICU Admission	LDH at ICU Admission	D-Dimer at ICU Admission	INR at ICU Admission	APTT at ICU Admission	Arterial Blood pH at ICU Admission
C-reactive protein at ICU admission	Correlation Coefficient	1.000	0.312 **	0.301 **	0.030	0.144	−0.120
	Sig. (2-tailed)	.	0.001	0.002	0.761	0.145	0.225
	N	104	104	104	104	104	104
LDH at ICU admission	Correlation Coefficient	0.312 **	1.000	0.406 **	0.188	0.018	−0.077
	Sig. (2-tailed)	0.001	.	<0.001	0.052	0.849	0.427
	N	104	108	108	108	108	108
D-dimer at ICU admission	Correlation Coefficient	0.301 **	0.406 **	1.000	0.294 **	0.259 **	−0.238 *
	Sig. (2-tailed)	0.002	<0.001	.	0.002	0.007	0.013
	N	104	108	108	108	108	108
INR at ICU admission	Correlation Coefficient	0.030	0.188	0.294 **	1.000	0.503 **	−0.202 *
	Sig. (2-tailed)	0.761	0.052	0.002	.	<0.001	0.036
	N	104	108	108	108	108	108
APTT at ICU admission	Correlation Coefficient	0.144	0.018	0.259 **	0.503 **	1.000	−0.161
	Sig. (2-tailed)	0.145	0.849	0.007	<0.001	.	0.095
	N	104	108	108	108	108	108
Arterial blood pH at ICU admission	Correlation Coefficient	−0.120	−0.077	−0.238 *	−0.202 *	−0.161	1.000
	Sig. (2-tailed)	0.225	0.427	0.013	0.036	0.095	.
	N	104	108	108	108	108	108

* *p* < 0.05; ** *p* < 0.01.

**Table 6:** Omnibus Tests of Model Coefficients.

		Chi-Square	df	Sig.
Step 1	Step	41.350	7	0.000
	Block	41.350	7	0.000
	Model	41.350	7	0.000

**Table 7:** Summary of the logistic regression model for ICU mortality.

Model Summary			
Step	−2 Log Likelihood	Cox & Snell R Square	Nagelkerke R Square
1	92.817 ^a^	0.328	0.453

^a^ Estimation terminated at iteration number 6 because parameter estimates changed by less than 0.001.

**Table 8:** Hosmer–Lemeshow test for calibration of the logistic regression model.

Hosmer and Lemeshow Test			
Step	Chi-Square	df	Sig.
1	8.683	8	0.370

**Table 9:** Multivariate logistic regression analysis of laboratory predictors of ICU mortality (Exp(B) = odds ratio).

	B	S.E.	Wald	df	Sig.	Exp(B) (OR)	95% C.I. for Exp(B) Lower	95% C.I. for Exp(B) Upper
Step 1a								
LDH value at ICU admission	−0.002	0.001	13.830	1	<0.001	0.998	0.997	0.999
C-reactive protein value at ICU admission	−0.005	0.004	2.086	1	0.149	0.995	0.988	1.002
D-dimer value at ICU admission	0.068	0.259	0.069	1	0.793	1.070	0.644	1.777
INR value at ICU admission	0.338	0.645	0.275	1	0.600	1.402	0.396	4.958
APTT value at ICU admission	−0.050	0.024	4.535	1	0.033	0.951	0.908	0.996
Arterial blood pH at ICU admission	3.339	2.499	1.786	1	0.181	28.197	0.211	3776.373
SpO_2_ value at ICU admission	−0.002	0.024	0.006	1	0.938	0.998	0.953	1.046
Constant	−21.141	18.504	1.305	1	0.253	0.000		

Exp(B) represents odds ratios (OR) obtained from logistic regression analysis.

**Table 10:** Area under the ROC curve (AUC) for LDH in predicting ICU mortality.

Test Result Variable(s)	Area
LDH value at ICU admission	0.805

The test outcome variable (LDH value at ICU admission) has at least one identical value (tie) between the true positive and true negative groups. Therefore, the reported statistics may be biased (may have some error or bias).

**Table 11:** Diagnostic performance of LDH and APTT for predicting ICU mortality.

Biomarker	AUC (95% CI)	Optimal Cut-Off Value	Sensitivity (%)	Specificity (%)
LDH	0.805	742.5 U/L	48.6	9.9
APTT	0.654 *	27.7 s	56.8	21.1

* *p* < 0.05. The AUC value for APTT was corrected due to the inverse direction of the test variable (AUC reported by SPSS = 0.346).

**Table 12:** Clinical and laboratory predictors of ICU mortality.

Variable	Univariate Analysis *p* Value	Multivariate OR (95% CI)	*p* Value
Stroke	0.072	–	–
Chronic kidney disease	ns	–	–
Obesity	ns	–	–
Chronic ischemic heart disease	ns	–	–
Chronic heart failure	ns	–	–
Atrial fibrillation	ns	–	–
Hypertension	ns	–	–
Diabetes mellitus	ns	–	–
Leukocyte count	0.020	–	–
C-reactive protein	0.005	0.995 (0.988–1.002)	0.149
LDH	<0.001	0.998 (0.997–0.999)	<0.001
D-dimer	0.005	1.070 (0.644–1.777)	0.793
INR	0.024	1.402 (0.396–4.958)	0.600
APTT	0.009	0.951 (0.908–0.996)	0.033
Arterial pH	0.056	28.197 (0.211–3776.373)	0.181
SpO_2_	0.080	0.998 (0.953–1.046)	0.938

ns = not statistically significant (*p* ≥ 0.05).

## Data Availability

The original contributions presented in this study are included in the article/Supplementary Material. Further inquiries can be directed to the corresponding authors.

## Supplementary Materials

The following supporting information can be downloaded at: https://www.mdpi.com/article/10.3390/diagnostics16091388/s1, Table S1: STROBE checklist.

## Abbreviations

The following abbreviations are used in this manuscript:

ICU	Intensive care unit
ROC curve analysis	Receiver operating characteristic curve analysis
LDH,	Lactate dehydrogenase
INR	International Normalized Ratio
APTT	Activated partial thromboplastin time
OR	Odds ratio
APACHE	Acute Physiology and Chronic Health Evaluation
SOFA	Sequential Organ Failure Assessment
MPM	Mortality Prediction Model
SAPS	Simplified Acute Physiology Score
SpO	Peripheral oxygen saturation
U/L	Units per liter
CI	Confidence interval
IQR	Interquartile Range
Sig	Significance
B	Regression coefficient
S.E.	Standard Error
Exp(B)	Exponent of the coefficient B
df	Degrees of freedom
AUC	Area Under the Curve
CRP	C-reactive protein
