# Supplementary material for: Admission Biomarkers as Predictors of Mortality in Comatose Patients in the Intensive Care Unit: A Retrospective Pilot Study
Source: Diagnostics (Basel). 2026 May 3;16(9):1388. doi: 10.3390/diagnostics16091388 (PMC13163716; doi:10.3390/diagnostics16091388)
Supplement: Supplementary file 1 [file diagnostics-16-01388-s001.zip › diagnostics-4228502-supplementary.pdf]

Table S1. STROBE Statement—Checklist of items that should be included in reports of ***cohort studies***

	Item No	Recommendation	Page No
**Title and abstract**	1	(*a*) Indicate the study's design with a commonly used term in the title or the abstract	Title; Abstract
		(*b*) Provide in the abstract an informative and balanced summary of what was done and what was found	Abstract
**Introduction**			
Background/rationale	2	Explain the scientific background and rationale for the investigation being reported	Introduction (paragraphs 1–last)
Objectives	3	State specific objectives, including any prespecified hypotheses	Introduction (last paragraph)
**Methods**			
Study design	4	Present key elements of study design early in the paper	Methods 2.1 Study design
Setting	5	Describe the setting, locations, and relevant dates, including periods of recruitment, exposure, follow-up, and data collection	Methods 2.1; 2.2
Participants	6	(*a*) Give the eligibility criteria, and the sources and methods of selection of participants. Describe methods of follow-up	Methods 2.2 Participants and selection criteria
		(*b*) For matched studies, give matching criteria and number of exposed and unexposed	Not applicable
Variables	7	Clearly define all outcomes, exposures, predictors, potential confounders, and effect modifiers. Give diagnostic criteria, if applicable	Methods 2.2
Data sources/ measurement	8*	For each variable of interest, give sources of data and details of methods of assessment (measurement). Describe comparability of assessment methods if there is more than one group	*Methods 2.2*
Bias	9	Describe any efforts to address potential sources of bias	Discussion – Limitations
Study size	10	Explain how the study size was arrived at	Methods 2.2
Quantitative variables	11	Explain how quantitative variables were handled in the analyses. If applicable, describe which groupings were chosen and why	Methods 2.3 Statistical analysis
Statistical methods	12	(*a*) Describe all statistical methods, including those used to control for confounding	Methods 2.3 Statistical analysis
		(*b*) Describe any methods used to examine subgroups and interactions	Not applicable
		(*c*) Explain how missing data were addressed	Methods 2.2 (exclusion of incomplete records)
		(*d*) If applicable, explain how loss to follow-up was	Not applicable

		addressed	
		(*e*) Describe any sensitivity analyses	Not applicable
**Results**			
Participants	13*	(a) Report numbers of individuals at each stage of study—eg numbers potentially eligible, examined for eligibility, confirmed eligible, included in the study, completing follow-up, and analysed	Methods 2.2; Figure 1
		(b) Give reasons for non-participation at each stage	Methods 2.2 (exclusion of incomplete records)
		(c) Consider use of a flow diagram	Figure 1
Descriptive data	14*	(a) Give characteristics of study participants (eg demographic, clinical, social) and information on exposures and potential confounders	Results Table 1–3
		(b) Indicate number of participants with missing data for each variable of interest	Methods 2.2 (incomplete records excluded)
		(c) Summarise follow-up time (eg, average and total amount)	Not applicable (outcome = ICU mortality during hospitalization)
Outcome data	15*	Report numbers of outcome events or summary measures over time	Results (mortality outcome)
Main results	16	(*a*) Give unadjusted estimates and, if applicable, confounder-adjusted estimates and their precision (eg, 95% confidence interval). Make clear which confounders were adjusted for and why they were included	Results Table 9; Table 12
		(*b*) Report category boundaries when continuous variables were categorized	Table 11 (cut-off values LDH, APTT)
		(*c*) If relevant, consider translating estimates of relative risk into absolute risk for a meaningful time period	Not applicable
Other analyses	17	Report other analyses done—eg analyses of subgroups and interactions, and sensitivity analyses	Table 5; Table 10–11; Figure 2–4
**Discussion**			
Key results	18	Summarise key results with reference to study objectives	Discussion (first paragraphs)
Limitations	19	Discuss limitations of the study, taking into account sources of potential bias or imprecision. Discuss both direction and magnitude of any potential bias	Discussion – Limitations of the study
Interpretation	20	Give a cautious overall interpretation of results considering objectives, limitations, multiplicity of analyses, results from similar studies, and other relevant evidence	Discussion (main body)
Generalisability	21	Discuss the generalisability (external validity) of the study results	Discussion – Limitations; Future

			research directions
**Other information**			
Funding	22	Give the source of funding and the role of the funders for the present study and, if applicable, for the original study on which the present article is based	No funding

*Give information separately for exposed and unexposed groups.

Note: An Explanation and Elaboration article discusses each checklist item and gives methodological background and published examples of transparent reporting. The STROBE checklist is best used in conjunction with this article (freely available on the Web sites of PLoS Medicine at http://www.plosmedicine.org/, Annals of Internal Medicine at http://www.annals.org/, and Epidemiology at http://www.epidem.com/). Information on the STROBE Initiative is available at http://www.strobe-statement.org.
